# Cathepsin L-mediated resistance of paclitaxel and cisplatin is mediated by distinct regulatory mechanisms

**DOI:** 10.1186/s13046-019-1299-4

**Published:** 2019-08-01

**Authors:** Yifan Zhao, Xiao Shen, Ying Zhu, Anqi Wang, Yajie Xiong, Long Wang, Yao Fei, Yan Wang, Wenjuan Wang, Fang Lin, Zhongqin Liang

**Affiliations:** 1 Department of Pharmacology, College of Pharmaceutical Sciences, Soochow University, Ren’ai Road 199, Suzhou, 215000 China; 2 Department of neurosurgery, Suzhou Kowloon Hospital, Shanghai Jiaotong University School of Medicine, Suzhou, 215000 China; 3 Department of Pharmacy, Children’s Hospital of Soochow University, Suzhou, 215000 China

**Keywords:** CTSL, Drug resistance, TGF-β, smad3, Egr-1, CREB

## Abstract

**Background:**

Cathepsin L (CTSL) is a cysteine protease known to have important roles in regulating cancer cellular resistance to chemotherapy. However mechanism underlying which regulates CTSL-mediated drug resistance remain largely unknown.

**Methods:**

We used NSCLC cell lines: A549, A549/TAX (paclitaxel-resistant), A549/DDP (cisplatin-resistant), H460 and PC9 cells, to evaluate CTSL and drug resistance changes. Tumor specimens from 53 patients with NSCLC and Xenograft models was also utilized to explore the regulatory relationship of CTSL, TGF-β, Egr-1 and CREB.

**Results:**

TGF-β and smad3 were overexpressed only in A549/TAX cells, silencing TGF-β or smad3 in A549/TAX cells decreased the expression of CTSL and enhanced their sensitivity to paclitaxel. Smad3 binds to the Smad-binding-element(SBE) of the CTSL promoter, resulting in increased activity of the CTSL promoter and subsequent CTSL. Egr-1 and CREB were overexpressed only in A549/DDP cells, and silencing Egr-1 or CREB reduced the expression of CTSL and increased cisplatin cytotoxicity. CREB could affect the activity of the CTSL promoter by binding to it. And the potential regulatory factors of CTSL were consistent in vivo and in human lung cancer. These different regulatory mechanisms of CTSL-mediated drug resistance exist in two other NSCLC cell lines.

**Conclusion:**

CTSL-mediated drug resistance to paclitaxel and cisplatin may be modulated by different mechanisms. The results of our study identified different mechanisms regulating CTSL-mediated drug resistance and identified smad3 as a novel regulator of CTSL.

**Electronic supplementary material:**

The online version of this article (10.1186/s13046-019-1299-4) contains supplementary material, which is available to authorized users.

## Background

Cathepsin L (CTSL), a cysteine protease that belongs to the papain-like family, has been found to be overexpressed in several types of human carcinomas arising from the lung, ovary, cervix, breast and colon [1, 2]. CTSL plays an important role in tumor occurrence, development, and metastasis [3, 4]. In recent years, it has been observed CTSL is highly correlated with drug resistance. In our laboratory, we have indicated that CTSL upregulation-induced EMT contributes to paclitaxel and cisplatin resistance in A549 cells [5]. CTSL inhibition in drug-resistant cells not only facilitates the induction of senescence, it also prevents drug resistance [6, 7]. The upregulation of CTSL in human cancers contributes to tumor growth and survival, and to resistance of PC9 cells to the chemotherapeutic drug gefitinib [8]. Additionally, silencing CTSL can prevent the drug resistance of tumor cells, reduce the proliferation of ovarian cancer cells, weaken the cell invasion and migration properties, and increase the sensitivity of cells to paclitaxel [9–11]. These studies suggested that CTSL may be a key target of drug resistance in cancer. At present, the mechanisms modulating CTSL-mediated drug resistance are unclear.

Transforming growth factor-β (TGF-β) is a multifunctional cytokine that plays an important roles in regulating the dynamic balance of cells and tissues, including proliferation, differentiation, migration, cell survival and angiogenesis [12]. Recent studies indicate that TGF-β may mediate drug resistance of tumor cells by promoting invasion and migration via EMT [13–15]. Previous studies in our laboratory have indicated that downregulation of CTSL can significantly inhibit invasion and migration in A549 cells through a TGF-β-mediated EMT pathway [16], but the regulatory mechanism between CTSL and TGF-β is unknown. Recent studies suggest the presence of a feedback mechanism between cathepsin B and TGF-β which regulates the invasion and migration of melanoma cells [17]. Nevertheless, the exact regulatory relationship between TGF-β and CTSL has not been determined, and whether TGF-β can regulate CTSL-mediated drug resistance remains unknown.

The smad signaling pathway is the most important signal transduction pathway in TGF-β signal transduction [18–20]. Smad3 is a member of the smad protein family and has been reported be involved in drug resistance, as its inhibition may reverse multidrug resistance in breast cancer cells [21]. Smad3 may specifically recognize and combine with the DNA sequence “GTCTAGAC” in promoter regions, which is called the smad binding element (SBE) [22]. As smad3 recognizes and combines with SBE, the presence of SBE in the CTSL promoter prompted us to investigate the potential role of smad3 in CTSL-mediated drug resistance. Our subsequent results suggest that TGF-β may be involved in regulating CTSL-mediated paclitaxel resistance in A549 cells, but not in CTSL-mediated cisplatin resistance in A549 cells. The mechanisms regulating CTSL-mediated paclitaxel and cisplatin resistance may be different, and there may be other signaling mechanisms regulating cisplatin resistance in A549 cells.

Cisplatin is a platinum chemotherapeutic agent reported to promote the expression of early growth response protein-1 (Egr-1) through the activation of the Egr-1 promoter [23, 24]. Egr-1 is a member of the early gene family, and can be induced by several physicochemical factors including ionizing radiation, drugs, and hypoxia. Recent studies suggest that Egr-1 is also involved in drug resistance in cancer cells [25–27]. Importantly, Ishidoh et al.’s research revealed a new mechanism of regulating CTSL where Egr-1 family proteins are involved in the activation of the CTSL gene in SR-3Y1-2 cells [28]. Sriraman et al.’s research indicated that Egr-1 could modulate the transcription of CTSL by binding differential sequences of the CTSL promoter. Sriraman’s research not only revealed the presence of an Egr-1 binding sequence, it also identified the presence of a CREB binding sequence in the CTSL promoter [29]. cAMP regulatory element-binding protein (CREB) is an important nuclear transcription factor which plays a key role in the regulation of gene transcription and cell development and survival. Moreover, Omira et al.’s study indicates that CREB may be involved in modulating the expression of CTSL by binding to its promoter [30], but whether Egr-1 and CREB may regulate CTSL-mediated drug resistance in A549 cells is unknown.

Based on the involvement of CTSL in resistance to chemotherapy, in this study we investigated the regulatory mechanisms of CTSL-mediated resistance to paclitaxel and cisplatin in A549 cells. We demonstrate that: (1) TGF-β promotes the expression of CTSL through the TGF-β/smad signaling pathway and smad3 binds with the CTSL promoter to increase CTSL transcription in paclitaxel resistance. (2) Egr-1 and CREB mediate the expression of CTSL to regulate cisplatin resistance, and CREB increases the transcriptional activity of CTSL through binding with the CTSL promoter. Thus, CTSL may represent a novel therapeutic target for reinforcing the efficacy of cancer chemotherapy.

## Materials and methods

### Cell lines and culture

The human lung cancer cell lines A549, PC9 and H460 were purchased from the Type Culture Collection of the Chinese Academy of Sciences, Shanghai, China. A549/TAX (paclitaxel-resistant A549 cells) and A549/DDP (cisplatin-resistant A549 cells) were purchased from shanghai MEIXUAN Biological Science and Technology Co, Ltd. All cells were cultured in high DMEM medium supplemented with 10% fetal bovine serum and penicillin (100 U/mL)/streptomycin (100 U/mL). A549/TAX and A549/DDP cells were cultured with complete medium with 200 ng/mL paclitaxel or 20 ng/mL cisplatin to maintain their drug resistance. Cells were maintained at 37 °C in a humidified atmosphere containing 5% CO_2_.

### Reagents and antibodies

Paclitaxel and cisplatin were purchased from Suzhou Kowloon Hospital (Suzhou, China); Cell Counting Kit-8 were purchased from Keygen Biotech (Nanjing, China); The antibodies to TGF-β, smad3, p-smad2, p-smad3 and GAPDH were purchased from RUIYING Technology (Suzhou, China); anti-Egr-1 was obtained from Santa Cruz Biotechnology Inc. (Santa Cruz, CA, USA); anti-CREB was obtained from Cell Signaling Technology (Danvers, MA, USA); anti-CTSL was purchased from Abcam (Abcam, USA). All of the cell culture media and other reagents were from Invitrogen.

Human NSCLC tissue samples (*n* = 53) were collected from surgically resected specimens form patients in the Affiliated Hospital of Jiangsu University, Zhenjiang, China with written informed consent of patients.

### Cytotoxicity assay

Cell Count Kit-8 assay (CCK8) was used to measure the viability and proliferation of cells. Cells were seeded into 96-well plates at a density of 10^4^ cells per well. The cells were then cultured for 24 h in 100 μL of DMEM complete medium. After pretreatment with different concentrations of paclitaxel or cisplatin for 24 h, 10 μL of CCK8 solution was added to each well and incubated for 1 h at 37 °C. The optical density was measured at 570 nm. All assays were performed in triplicate.

### Western blot assay

Cells were harvested using a plastic scraper and then washed twice with cold PBS. Afterward, the cells were homogenized in lysis buffer. Proteins in the lysates were quantified using the BCA™ Protein Assay Kit (Thermo Scientific, Rockford, USA). At the end of centrifugation, cell lysates were collected and protein concentration of cell lysates were measured. Proteins (50 μg) were resolved by SDS–polyacrylamide gel electrophoresis and transferred to PVDF membrane from Bio-Rad (Hercules, CA, USA). The blots were then incubated with primary antibodies in 5% skimmed milk powder/Tris-buffered saline Tween-20 at 4 °C overnight, followed by incubation with secondary antibodies at room temperature for 1 h. Then the protein signals were detected using the Odyssey Infrared Imaging System (Li-COR Biosciences, Lincoln, NE, USA).

### siRNA and plasmid transfection

siRNAs targeting TGF-β, samd3, Egr-1, CREB and the nontargeting siRNA were purchased from GenePharma (Shanghai, China). Luciferase reporter plasmids were synthesized by Suzhou Golden Wisdom Biological Technology Co., Ltd. Cells in exponential phase of growth were plated in 6-well cell culture plates at 1 × 10^5^ cells/well, grown for 24 h, and then transfected with siRNA using lipofectamine 2000 (Invitrogen) according to the manufacturer’s protocol. The chain of interference for transfection cells includes in Table 1.

**Table 1:**
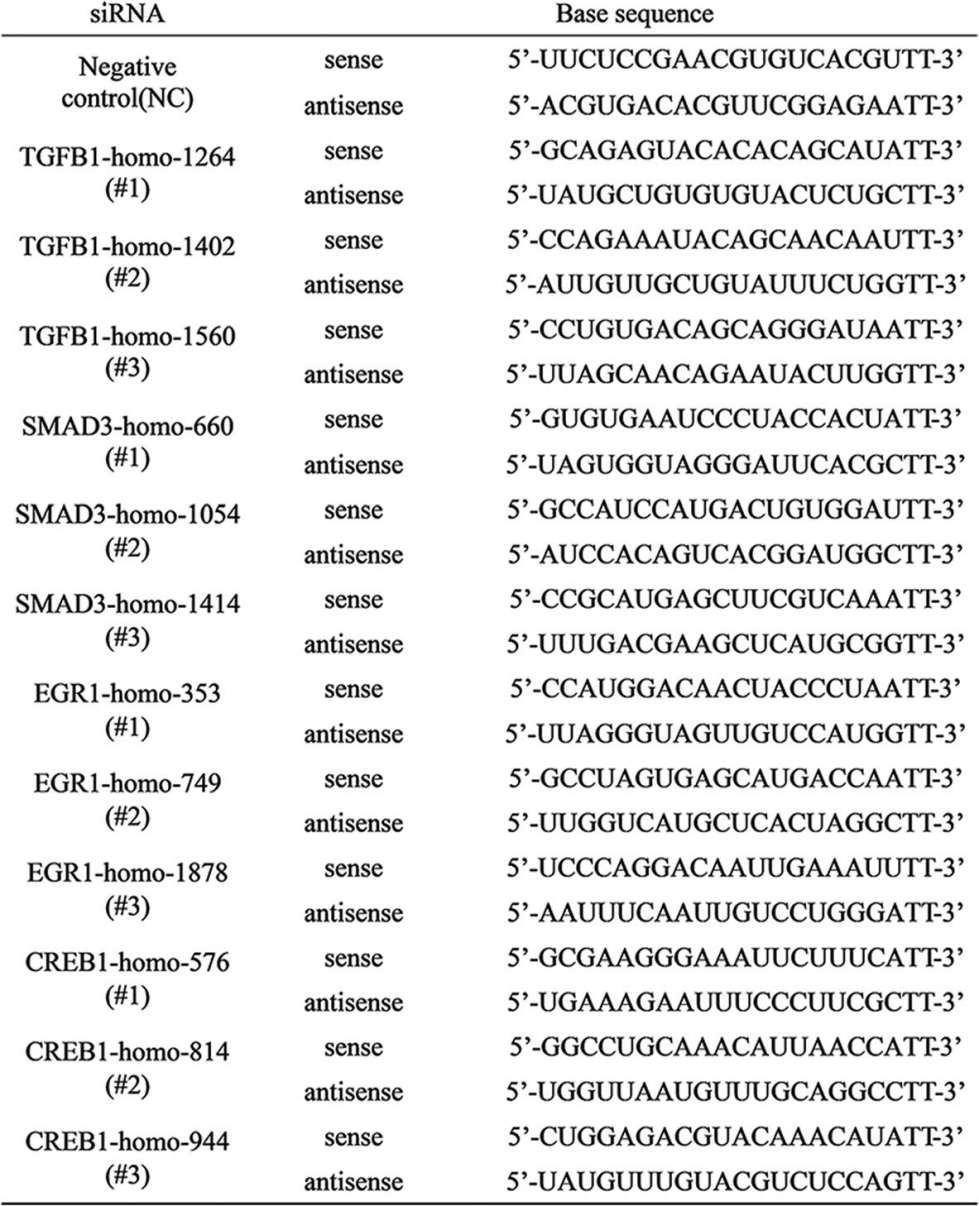
The sequence of siRNA for transfection cells

### Immunofluorescence staining

Cells were seeded into 24-well plates at density of 300 cells per plate, then were treated as required for the experiment. Culturing for 24 h, the cells were fixed with methanol for 10 min at 4 °C and permeabilized for 10 min with 0.1% Triton X-100. The cells were then incubated for 1 h in blocking buffer (1% BSA and 0.1% Triton X-100) at 4 °C. For immunofluorescence (IF), the cells were incubated with antibodies against CTSL and smad3 at 4 °C overnight. The cells were then incubated with the appropriate biotinylated secondary antibodies for 1 h. Alexa Fluor 488 (Molecular Probes, 1:500) antibodies were used as tertiary antibodies for 1 h. And then the cells were counterstained with 0.5 ng/mL DAPI for 15 min at room temperature. Coverslips were mounted on slides with VECTASHIELD Mounting Medium for fluorescence and analyzed by confocal microscopy.

### Apoptosis assay

Apoptosis was measured using an Annexin V-(FITC)/PI apoptosis detection kit (Keygen, Nanjing, China). Briefly, the cells were cultured into the 6-well plates for 24 h, then were treated as required for the experiment. Culturing for 24 h, collecting the cells by Trypsin without EDTA into a centrifuge tube, mixed it with 500 uL of binding buffer, and added 5 uL Annexin V-FITC and 5 uL PI reagent into every tube, then the cells were incubated with the reagent for 15 min at room temperature in the dark. At the end of incubation, the cells were analyzed by a FACSCalibur flow cytometer.

### Construction of luciferase reporter gene detection carrier

Using pGL4 enhancer carrier to construct the luciferase reporter gene of smad3 binding region (SBE) (5′-AGACAGACAGACAGACGTCTGTCTGTCTGTCT-3 ‘) and the luciferase reporter gene of CREB binding region in the CTSL promoter (5’-CCCAGCTCTGGGACAGTCAGTAAACAAGCCACGAACCGCGCCAGGGATCAGAGCACCCAGAGTCCCCGCCCAGCTGCCGGCACAGCCAATCGCAGCGCAGCCAGGCGGCGGGGCGGTGCCGGCCGAACCCAGACCCGAGGTTTTAGAAGCAGAGTCAGGCGAAGCTGGGCCAGAACCGCGACCTCCGCAACCTTGAGCGGCATCCGTGGAGTGCGCCTGCGCAGCTACGACCGCAGCAGGAAAGCGCCGCCGGCCAGGCCCAGCTGTGGCCGGACA-3 ‘). The luciferase reporter gene carriers were synthesized by Suzhou Golden Wisdom Biological Technology Co., Ltd.

### Luciferase reporter assay

A549 and A549/TAX cells were cultured with or without silencing of smad3 expression were transfected with pGL4-SBE-Luciferase plasmid, and A549 and A549/DDP cells were cultured with or without silencing of CREB expression were transfected with pGL4-CREB-Luciferase plasmid using lipofectamine 2000. Forty-eight hours later, the cells were collected in the special lysate, added 50 uL β-gal substrate into 10 uL protein sample at 37 °C in the dark, and added 10 uL Luciferase substrate into another 10 uL protein sample, using the Luciferase Reporter Assay System and Iuminometer to detect the luciferase activity.

### Chromatin immunoprecipitation (CHIP) assay

A549 and A549/TAX cells were seeded in 100 mm cell plate, after culturing for 24 h, CHIP assay was conducted. CHIP was performed with rabbit smad3 antibody according to the manufacturer’s protocol (Millipore, USA). Then use the TIANGEN kit to extract DNA according to the instructions.

PCR was used to test the performance of the CHIP. The gene of CTSL was amplified by touch-down PCR. The primer of CTSL gene was synthetized by shanghai Abm Co., Ltd. The primer sequences for CTSL gene were as follows: CTSL forward primer 5′-GTGACTGGTTGAGCGGGCAG-3′ and CTSL reverse primer 5′-GCCACACACTGGCTGTAGCG-3′. The amplification products were electrophoresed in 1.0% agarose gel and DNA sequencing was conducted by Sangon Biotech (Shanghai) Co., Ltd. (China).

### Animal experiments

This study was carried out in accordance with the principles of the Declaration of Helsinki and approved by the Ethics Committee of Soochow University Medical School. Five week-old male nude (BALB/c) mice (Animal Experiment Center of Soochow University, Suzhou, China) were used in the experiments. A549 cells (5 × 10^7^ cells/0.1 mL medium/mouse) were injected into the mice subcutaneously to generate the mouse models. Xenografts were allowed to grow to approximately 100 mm^3^ over 2 weeks and randomly divided into three groups (*n* = 5 in each group) as follows: control (100 μL, saline solution), paclitaxel (15 mg/kg) and cisplatin (15 mg/kg). Paclitaxel was administered by intraperitoneal injection every 3 d for 3 weeks and cisplatin was administered by intraperitoneal injection every 6 d for three times. At the end of the experiments, tumor tissues were harvested from these mice for immunohistochemistry or western blot analysis.

### Immunohistochemical staining

Immunostaining was conducted using the Vectastain ABC kit (Vector) in accordance with the manufacturer’s instructions. Briefly, the slides were deparaffinized, rehydrated, and treated with a citric acid solution to prepare them for immunohistochemical studies. After blocking endogenous peroxidase activity by preincubation in 3% hydrogen peroxide solution, the slides were incubated in blocking solution (PBS, 3% bovine serum albumin) and then sequentially incubated with primary antibodies. The sections were counterstained with hematoxylin (Sigma) for nuclear staining. Negative control slides without primary antibodies did not exhibit nonspecific staining. The slides were independently evaluated by two investigators who were blinded to the experimental data.

### Statistical analysis

Data were expressed as the mean ± SD. At least three independent experiments were performed. Differences in measured variables between the experimental and control groups were assessed using Student’s t-test. *P* values less than 0.05 were considered statistically significant. All analyses were performed using GraphPad Prism 5.0.

## Results

### TGF-β participates in the regulation of CTSL mediated paclitaxel resistance through activating TGF-β/smad signaling pathway

**Fig. 1:**
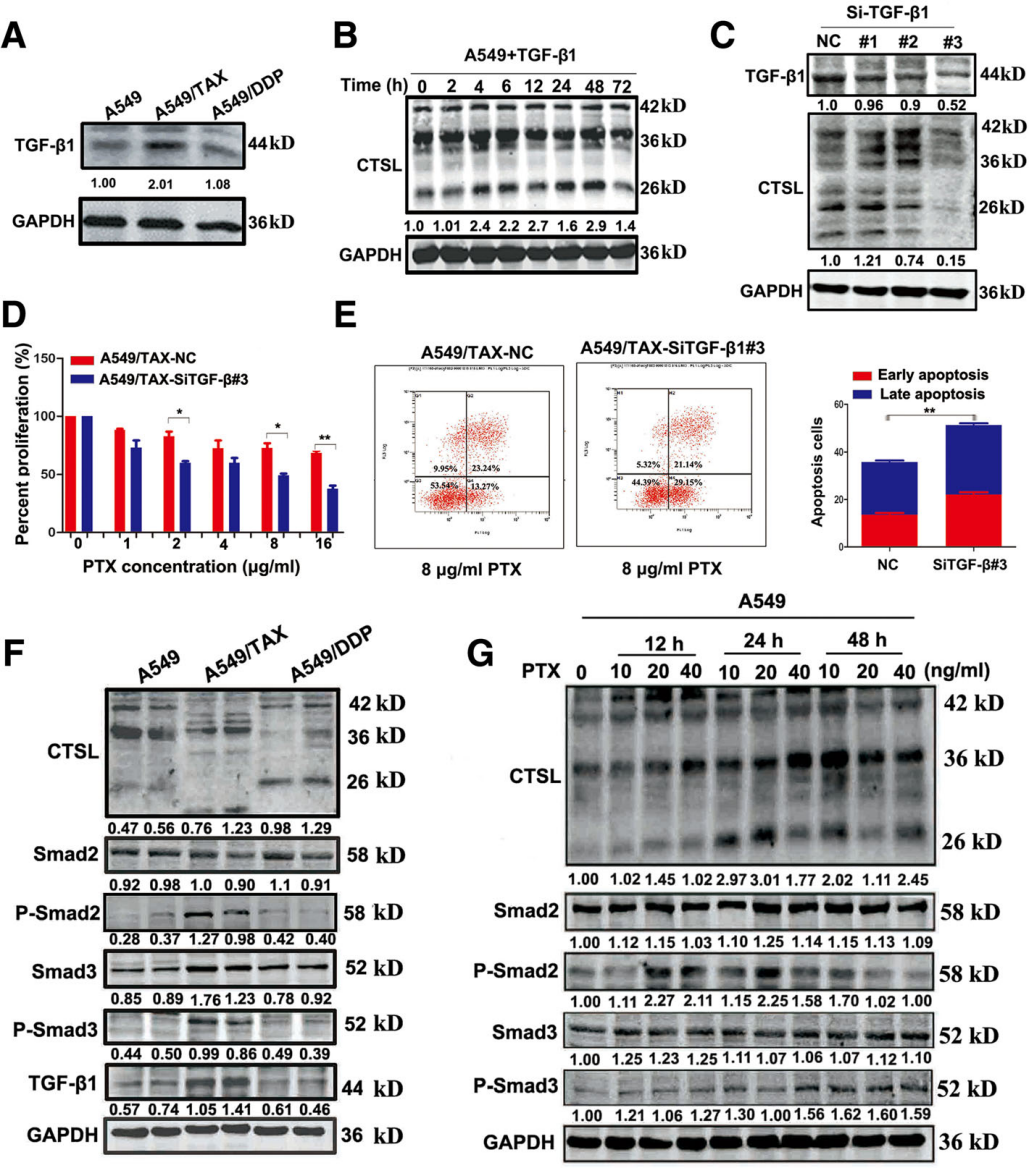
TGF-β participates in the regulation of CTSL mediated paclitaxel resistance in A549 cells. **a** Western blot determined the expression of TGF-β of three cell lines. **b** A549 cells were treated with 10 μM TGF-β and harvested at different times, western blot was performed to detect the expression of CTSL protein level. **c** Western blot detected the expression of TGF-β and CTSL of A549/TAX cells transfected with TGF-β siRNAs targeting the human TGF-β sequence or the control siRNA. **d** and **e** CCK8 and flow cytometry assays were conducted to measure the change of paclitaxel resistance of A549/TAX cells. **f** Western blot was conducted to measure the expression level of TGF-β/smads signaling pathway associated proteins of three cell lines. **g** A549 cells were treated with different concentration of paclitaxel and harvested at 12 h, 24 h and 48 h, and western blot detected the expression level of TGF-β/smads signaling pathway associated proteins. At least three independent experiments were performed. *P < 0.05, **P < 0.01 and ***P < 0.001 compared with control

CTSL was overexpressed in drug-resistant cell lines (Additional file 1: Figure S1). Decreased expression of CTSL in lung cancer cells has been reported to inhibit EMT mediated by TGF-β, and CTSL increases after the treatment of cells with TGF-β [16]. Nevertheless, the regulatory relationship between CTSL and TGF-β remain largely unknown. As a first step to investigate the relationship between CTSL and TGF-β, western blot was used to detect the expression of CTSL and TGF-β in A549, A549/TAX and A549/DDP cells. The results showed that TGF-β was highly expressed in A549/TAX cells (Fig. 1a), but not in A549/DDP cells, indicating that the regulatory mechanisms of CTSL-mediated paclitaxel and cisplatin resistance may be different. Stimulating A549 cells with 10 μM TGF-β, CTSL was substantially increased at 4 h (Fig. 1b). Overall, to underscore the role of TGF-β in regulating CTSL-mediated drug resistance to paclitaxel, the TGF-β siRNA was used in A549/TAX cells. As shown, knockdown of TGF-β reduced the expression of CTSL, decreased cell proliferation, increased apoptosis, and enhanced the sensitivity of A549/TAX cells to paclitaxel (Fig. 1c-e). These results indicate that TGF-β may be involved in regulating CTSL-mediated drug resistance to paclitaxel in A549 cells. Smad signaling pathway is the most classic pathway of TGF-β [18]. In addition, to determine whether the effect of TGF-β was dependent on the smad signaling pathway, the expression of smad-associated proteins were detected by western blot. Compared with A549 cells, robust phosphorylation of smad2 and smad3 was detected only in A549/TAX cells (Fig. 1f), suggesting the TGF-β/smad signaling pathway was activated. When A549 cells were treated with a gradient concentration of paclitaxel at 12, 24 and 48 h, the phosphorylation levels of smad2 and smad3 were clearly increased (Fig. 1g), this effect was not caused by the cytotoxicity of paclitaxel. Most notably, SB431542, a TGF-β II receptor inhibitor, markedly decreased the expression of active CTSL in A549/TAX cells (Additional file 2: Figure S2A). Thus, our data indicated that TGF-β may modulate CTSL-mediated paclitaxel resistance through activating TGF-β/smad signaling pathway.

### Smad3 regulates CTSL mediated drug resistance through binding with the CTSL promoter

**Fig. 2:**
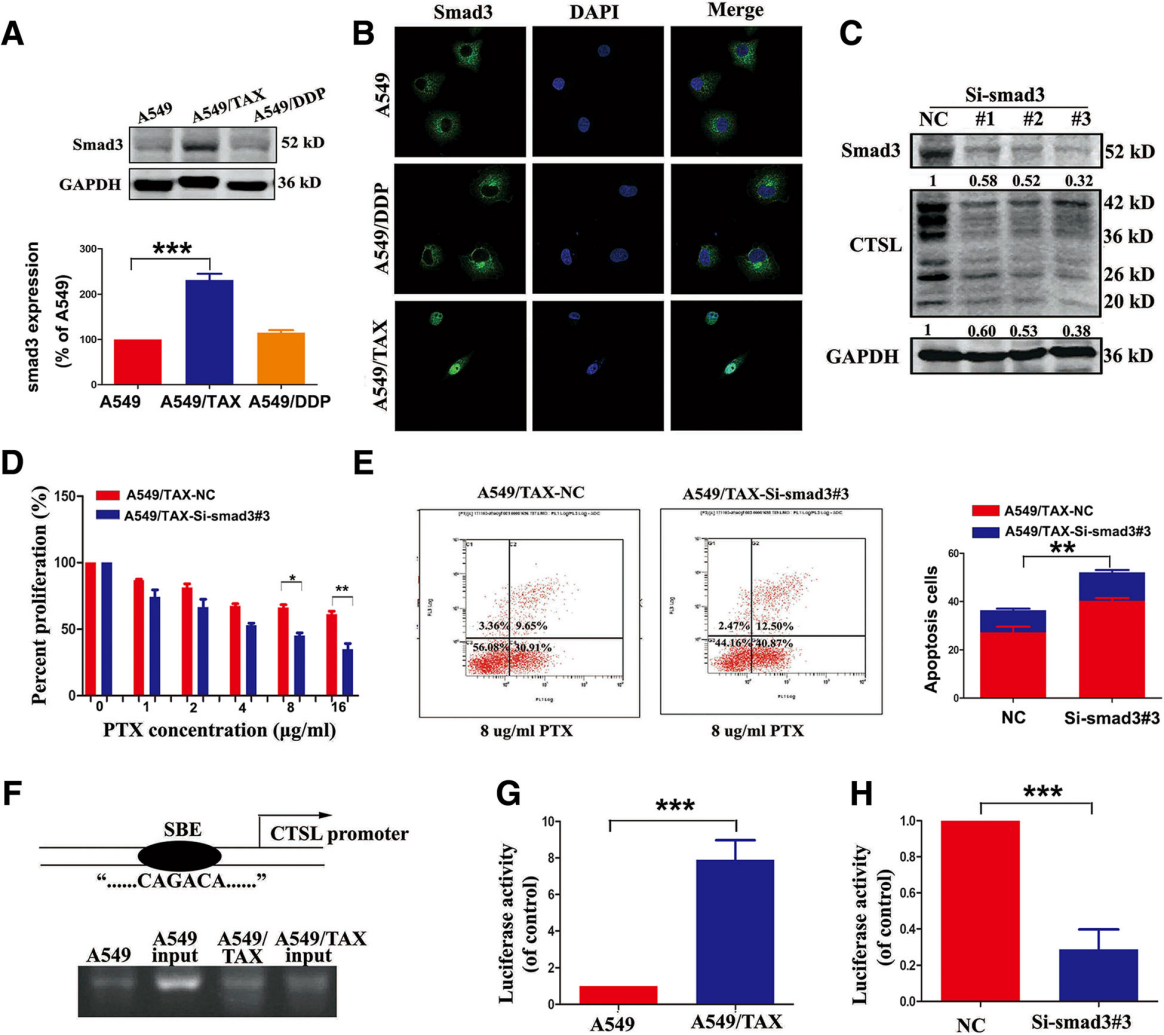
Smad3 regulates CTSL mediated drug resistance through binding with the CTSL promoter. **a** and **b** Western blot and immunofluorescence analysis were adopted to determine the expression level of smad3 in A549, A549/TAX and A549/DDP cells. **c** Western blot detected the expression of smad3 and CTSL in A549/TAX cells transfected with smad3 siRNAs targeting the human smad3 sequence or the control siRNA. **d** and **e** CCK8 and flow cytometry assay determined the change of paclitaxel resistance in A549/TAX cells transfected with smad3 siRNAs and the control. **g** CHIP assay was conducted to detect the interaction between smad3 and CTSL promoter. **h** Luciferase assay was performed to detect CTSL promoter activity of A549 and A549/TAX cells. **i** Luciferase assay was used to detect CTSL activity of A549/TAX cells transfected with smad3 siRNAs and the control. At least three independent experiments were performed. *P < 0.05 and **P < 0.01 compared with control

In Fig. 1, we also observed that smad3 was highly expressed, and nuclear localization of smad3 only appeared in A549/TAX cells (Fig. 2a-b). Inhibition of smad3 has been reported to inhibit the proliferation, invasion and migration of cancer cells. To determine whether smad3 could be a regulator of CTSL-mediated drug resistance to paclitaxel, the smad3 siRNA was synthesized, then transfected into A549/TAX cells. The results showed that the expression of active CTSL was decreased, cell proliferation was decreased and apoptosis was increased, enhanced the paclitaxel sensitivity in A549/TAX cells(Fig. 2c-e). Smad3 is highly conserved in different species of mammals. Since nuclear localization of smad3 was observed in A549/TAX cells, we asked whether smad3 could bind with the SBE of CTSL promoter to play a regulatory role in promoter activity to modulate CTSL-mediated paclitaxel resistance. CHIP assay was conducted to further investigate the relationship of smad3 with CTSL. The PCR results showed that smad3 could bind to the promoter of CTSL, and the binding level in A549/TAX cells was greater than in A549 cells (Fig. 2f). We also found that the activity of the CTSL promoter in A549/TAX cells was notably higher than in A549 cells (Fig. 2g). Inhibition of smad3 in A549/TAX cells decreased the activity of the CTSL promoter compared with controls (Fig. 2h). The results above showed that smad3 may regulate CTSL-mediated drug resistance to paclitaxel through binding with the SBE of the CTSL promoter to increase CTSL transcription.

### Egr-1 and CREB were involved in cisplatin resistance mediated by CTSL

**Fig. 3:**
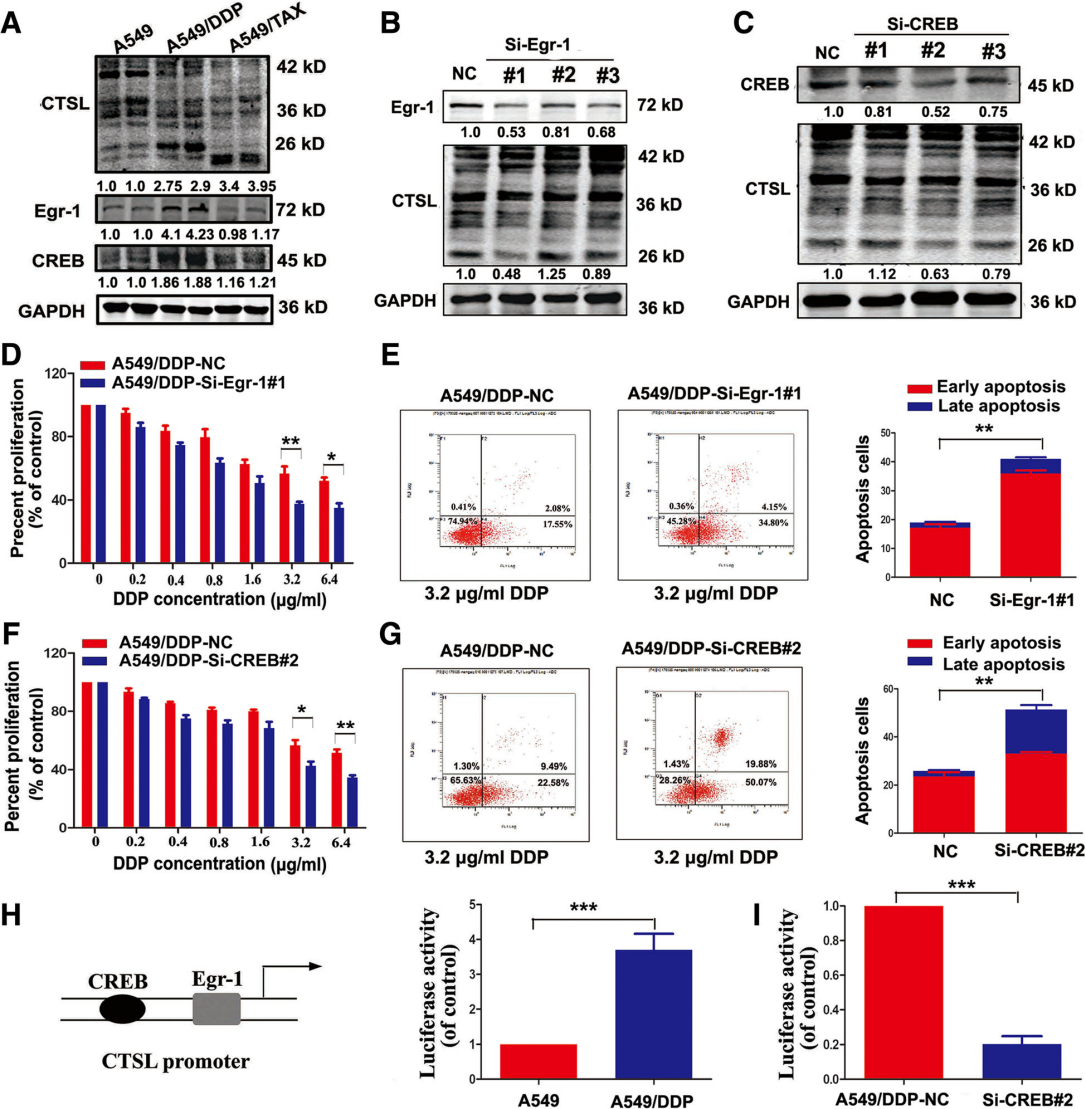
Egr-1 and CREB were involved in cisplatin resistance mediated by CTSL. **a** Western blot was conducted to measure the expression level of Egr-1 and CREB in A549, A549/TAX and A549/DDP cells. **b** Silence of Egr-1 in A549/DDP cells by transfecting Egr-1 siRNA, and then western blot was used to detect the expression level of Egr-1 and CTSL. **d** and **e** CCK8 and flow cytometry assay were conducted to determine the change of cisplatin resistance in A549/DDP cells transfected with Egr-1 siRNAs and the control. **c** Western blot was adopted to detect the expression of CREB of A549/DDP cells transfected with CREB siRNAs and the control. **f** and **g** CCK8 and flow cytometry assay were performed to determine the change of cisplatin resistance of CREB-silenced A549/DDP . **h** Luciferase assay was used to detect CTSL promoter activity in A549 and A549/DDP cells. **i** Luciferase assay was performed to detect CTSL promoter activity in A549/DDP cells transfected with CREB siRNAs and the control. At least three independent experiments were performed. *P < 0.05, **P < 0.01 and ***P < 0.001 compared with control

The above results indicate that TGF-β and smad3 do not participate in regulating CTSL-mediated drug resistance to cisplatin, there may be other regulatory factors which regulate CTSL in A549/DDP cells. Egr-1 and CREB regulate the expression of CTSL [29], but whether they can modulate CTSL-mediated drug resistance is not known. We first measured the expression of Egr-1 and CREB, and found they were highly expressed only in A549/DDP cells (Fig. 3a), and cisplatin treatment increased the expression of CTSL, Egr-1 and CREB in A549 cells (Additional file 2: Figure S2B-C). Then silencing Egr-1 significantly reduced the expression of active CTSL, weakened cell proliferation and increased apoptosis in the cisplatin-resistant cells, reinforcing the sensitivity of cisplatin (Fig. 3d-e). Additionally, knocked down the expression of CREB could reduce the expression of active CTSL, decrease cell proliferation, increase cell apoptosis, and decrease the drug resistance of A549/DDP cells (Fig. 3f-g). These results suggest that Egr-1 and CREB may be associated with CTSL-mediated drug resistance to cisplatin. Since CREB is a regulatory factor of CTSL transcription, we asked whether CREB regulates CTSL-mediated cisplatin resistance by affecting the transcription level of CTSL. Luciferase assay was used to detect the activity of the CTSL promoter, as shown, the activity of the CTSL promoter in A549/DDP cells was notably higher than in A549 cells (Fig. 3h), and inhibition of CREB in A549/DDP cells decreased CTSL activity compared with controls (Fig. 3i). Thus, CREB may regulate CTSL-mediated drug resistance to cisplatin through binding with the CTSL promoter to increase CTSL transcription.

### Smad3 and Egr-1 were positively correlated with CTSL in vivo

**Fig. 4:**
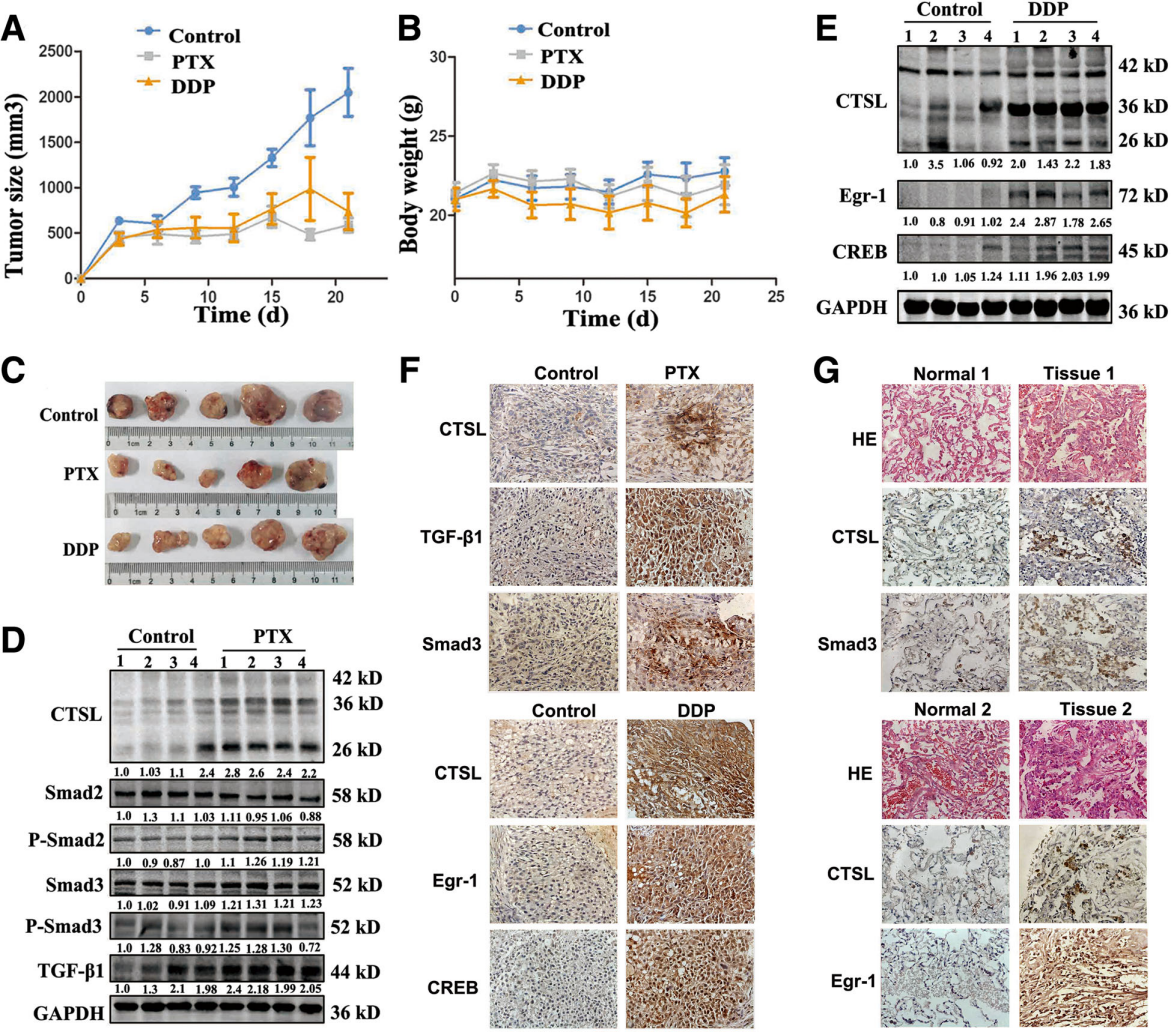
Smad3 and Egr-1 were positively correlated with CTSL in vivo. **a** A549 cells were injected to nude mice, which were treated with paclitaxel and cisplatin. The tumor size was measured using a Vernier caliper. **b** Body weight was determined using an electronic balance. **c** At the end of the treatment, the tumors were removed from the nude mice. And tumor lysates were resolved by SDS-polyacrylamide gel electrophoresis and subjected to western blot analysis using anti-CTSL, anti-smad3, anti-Egr-1, anti-CREB antibodies, respectively. GAPDH was used as a loading control. **d** and **e** Western blot was performed to detect the expression level of proteins. **f** and **g** Immunohistochemistry was performed to detect the expression of CTSL, smad3, Egr-1 and CREB in xenografted tumor and human NSCLC tissues. Photographs were obtained using a 40 × objective lens. At least three independent experiments were performed

Our in vitro results indicate that TGF-β and smad3 play crucial roles in regulating CTSL-mediated drug resistance to paclitaxel, and Egr-1 and CREB are involved in regulating CTSL-mediated drug resistance to cisplatin. We further investigated the regulatory mechanism of CTSL in vivo. The subcutaneous tumor xenograft model was established using A549 cells, and then treated with or not paclitaxel or cisplatin. Tumor volume and body weight were monitored, and tumor growth curves are shown in Fig. 4a and b. Subcutaneous injection of paclitaxel or cisplatin in A549 tumor-bearing mice induced significant inhibition of tumor growth for the entire 21-day study period (** *P* < 0.01; Fig. 4a). Mice in all groups showed no obvious signs of adverse effects with respect to body weight (Fig. 4b) and general activity. We next assessed levels of CTSL and associated proteins in three groups via Western blot and IHC. As shown, compared with the control groups, the expression of smad3 and active CTSL protein increased in paclitaxel-treatment groups, and paclitaxel induced the activation of the TGF-β/smad signaling pathway in vivo (Fig. 4d). CTSL, as well as Egr-1 and CREB levels, were also enhanced in the cisplatin-treatment groups (Fig. 4e). Taken together, these findings are consistent with in vitro results, and the immunohistochemistry findings were shown in Fig. 4f.

To further verify the correlations between these factors which may regulate CTSL in vivo, samples were extracted from 53 clinical lung cancer patients after homogenization of tumor samples and adjacent normal lung tissues. As shown (Fig. 4g), compared with normal tissue, the expression of CTSL in tumors tended to increase, and the expression of smad3 in these samples was proportional to the expression of CTSL. In 35 out of 53 tumors, the expression of smad3 in tumor tissue was higher than that of normal tissue, indicating potential drug resistance to paclitaxel. In 23 of the 53 tumors, Egr-1 expression was higher and positively correlated with CTSL. It is suspected that these patients may be drug resistance to cisplatin. However, due to lack of follow-up results, we can’t confirm that these patients were drug resistance to paclitaxel or cisplatin. However these results do indicate that smad3 and Egr-1 are positively correlated with CTSL in human lung cancer tissues.

### CTSL-mediated resistance of paclitaxel and cisplatin via distinct regulatory mechanisms in two other non-small lung cancer cells

**Fig. 5:**
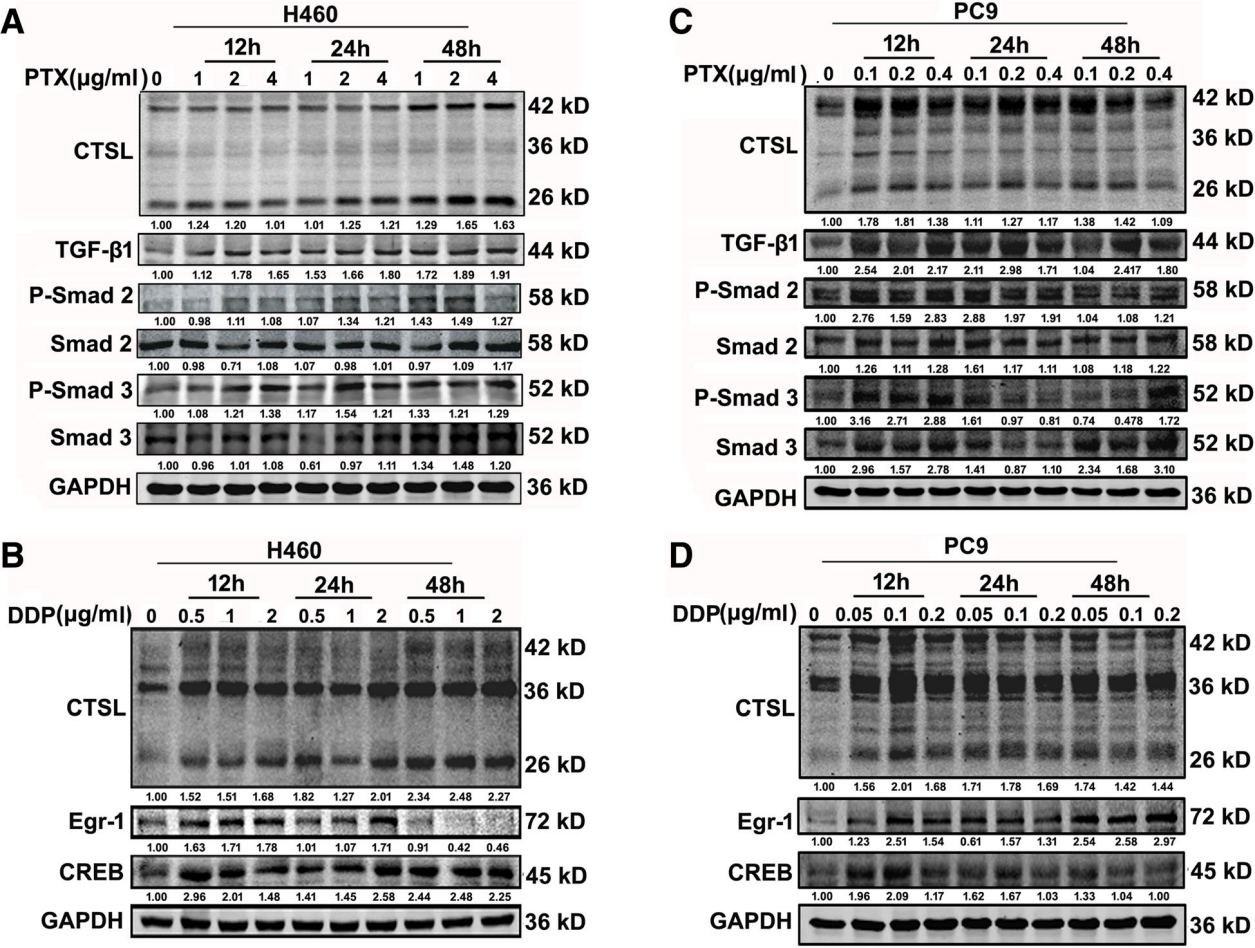
Cathepsin L-mediated resistance of paclitaxel and cisplatin via distinct regulatory mechanisms in two other non-small lung cancer. **a** and **c** PC9 and H460 cells were treated with different concentration of paclitaxel and harvested at 12 h, 24 h and 48 h, and western blot detected the expression level of CTSL and TGF-β/smad signaling pathway associated proteins. **b** and **d** PC9 and H460 cells were treated with different concentration of cisplatin and harvested at 12 h, 24 h and 48 h, and western blot detected the expression level of CTSL, Egr-1 and CREB. At least three independent experiments were performed

CTSL-mediated drug resistance to paclitaxel and cisplatin may be differently in A549 cells. To explore whether the differently regulatory mechanism exist in other non-small lung cancer cells: PC9 and H460. The IC50 of PTX and DDP in H460 and PC9 cells were shown in Additional file 3: Figure S3. We stimulated these cells with a gradient concentration of paclitaxel at 12, 24 and 48 h. The expression of active CTSL was clearly increased, the phosphorylation levels of smad2 and smad3 were elevated (Fig. 5a and c), and the TGF-β/smad signaling pathway was activated in these cells. We then treated the cells with different concentration of cisplatin. The results suggest that cisplatin treatment significantly increased the expression of active CTSL, Egr-1 and CREB in these cells (Fig. 5b and d). These results were consistent with the results in A549 cells.

## Discussion

In this study, we report previously unrecognized differential regulatory mechanisms for CTSL-mediated drug resistance. We provide evidence that the mechanisms regulating CTSL-mediated paclitaxel and cisplatin resistance differ. TGF-β modulates the expression of active CTSL through the activation of the TGF-β/smad signaling pathway to control the transcription of CTSL to regulate CTSL-mediated drug resistance to paclitaxel. We further demonstrate that the regulation of cisplatin resistance by CTSL is mediated through its effect on Egr-1 and CREB, which are known to be involved in the activation of the CTSL gene [27].

CTSL, a cysteine protease that is ubiquitous in lysosome, belongs to the papain family and plays an important role in the degradation of intracellular and intracellular proteins [31–33]. CTSL is usually stored in lysosomes in the form of a 42 KD precursor protein. Two CTSL short strands can be modified into active short chains of 36 KD and 26 KD. We detected the activity of CTSL in A549 cells, A549 paclitaxel-resistant cells and A549 cisplatin resistant cells. The expression of active CTSL was significantly increased in A549/TAX and A549/DDP cells, and there is an active 20 KD band of CTSL in A549/TAX cells, which suggests that CTSL may be further modified in A549/TAX cells. The role of these processing modifications merits further study.

Our study indicates that paclitaxel induced the expression of active CTSL by activating the TGF-β/smad signaling pathway in A549 cells. The activation of TGF-β/smad signaling pathway is only present in A549/TAX cells. We used SB431542, which is a TGF-β II receptor inhibitor, to inhibit the TGF-β/smad signaling pathway, and observed a similar decrease in active CTSL in A549/TAX cells, indicating that TGF-β/smad signaling pathway is indeed activated in A549/TAX cells (Fig. 3c).

We also observed that the expression of smad3 in A549/TAX cells was greater than that in the other two cell lines. Smad3 is an important transporter of TGF-β signaling pathway in A549/TAX cells. As shown in this study, smad3 may be involved in regulating CTSL-mediated paclitaxel resistance. Several studies have shown that smad3 is highly conserved in different species of mammals. There is a special “CAGACA” sequence in the MH1 domain, known as the smad binding element (SBE), which can selectively identify the promoter of target genes [22]. We identified a number of SBEs in the promoter of CTSL, and we are the first to report the combination of smad3 and the CTSL promoter by CHIP assay. Smad3 regulates the transcription and expression of CTSL by binding to the SBE on the CTSL promoter in the nucleus of A549/TAX cells, supporting a role for smad3 in regulating CTSL-mediated paclitaxel resistance.

Smad3 is associated with cell resistance, and we report that the expression of smad3 leads to CTSL-mediated drug resistance. In contrast, other studies report that the inhibition of smad3 promotes the invasion and migration of tumor cells, leading to drug resistance, while the overexpression of smad3 in tumor cells could inhibit cell proliferation, increase cell sensitivity to chemotherapeutic drugs, and improve drug resistance [34–37]. These results are contrary to the current data. Liu et al.’s study suggests that there are two different configurations of smad3, psmad3C and psmad3L, and the actions of the TGF-β signaling pathway with different configurations of smad3 may produce different tumor outcomes and prognoses [38, 39]. The current data indicate that smad3 promotes cellular resistance to paclitaxel, and it is likely that this effect may be related to cell types or specific effects of smad3.

In our study, we found that TGF-β and smad3 don’t regulate CTSL-mediated drug resistance to cisplatin in A549 cells, there were differently regulatory mechanism in regulating CTSL-mediated drug resistance to paclitaxel and cisplatin, maybe it was associated with the drug pharmacology. Cisplatin is a platinum chemotherapeutic agent, which was reported to stimulate the activity of Egr-1 promoter. As a member of the early gene family, Egr-1 has an important role in drug resistance in cancer cells and could regulate the expression of CTSL [24–27]. Previous studies in our laboratory have indicated that Egr-1 may be the target protein of mut-p53 in regulating CTSL mediated EMT under IR in NSCLC [40]. We indicated here that Egr-1 regulated CTSL-mediated cisplatin resistance in lung cancer by affecting the activity of CTSL promoter. In Fig. 3, we found silencing Egr-1 of A549/DDP cells couldn’t reverse the resistance completely, there may be other factors regulate the CTSL-mediated cisplatin resistance. CREB is an important nuclear transcription factor, which plays an important role in regulating gene transcription, cell development and survival. In the CTSL promoter, there has the presence of CREB binding sequence, we proved here that CREB could bind with the CTSL promoter to regulate CTSL-mediated drug resistance to cisplatin. Egr-1 and CREB may be the regulatory factors of CTSL. Additional further study is needed.

In our vivo experiments, we just found that TGF-β, smad3, Egr-1 and CREB were increased positively correlated with CTSL under the treatment of paclitaxel and cisplatin, we need to use some inhibitors of TGF-β, smad3, Egr-1 or CERB to further prove these factors play crucial roles in regulating CTSL-mediated drug resistance in the future. Inhibition of CTSL may enhanced the effect of paclitaxel or cisplatin to inhibit the growth of tumor [5, 16], especially for those patients with drug resistance. Inhibiting the upstream gene targets of CTSL, such as TGF-β, smad3, Egr-1 and CERB, may increase the activity of drugs in vivo. More studies should be done in further.

The development of drug resistance is a complex process involving multiple genes and associated pathways. The mechanism of CTSL mediated tumor resistance is also complex process, and the current results are preliminary. It is unclear whether the mechanisms regulating CTSL-mediated resistance to paclitaxel and cisplatin in other tumor cells is consistent with the mechanisms of drug resistance in A549 cells. Research with other cell lines is needed. We also report that micRNA-200c may regulate CTSL mediated paclitaxel resistance by forming a feedback loop with CTSL [41]. It is unknown whether other microRNAs could regulate CTSL-mediated drug resistance, and CTSL is also affected by additional regulatory factors such as NF-Y [42]. Additional study of factors modulating CTSL activity is needed.

In summary, our study identified differential regulatory mechanisms for CTSL-mediated drug resistance to paclitaxel and cisplatin. These findings expand current knowledge of drug resistance mechanisms and provide new insight into how CTSL-mediated drug resistance is regulated in A549 cells. It provides a theoretical and experimental basis for enhancing the efficacy of chemotherapy based clinical interventions.

## Conclusion

Our studies indicated that the mechanisms regulating CTSL-mediated paclitaxel and cisplatin resistance is differently. TGF-β and smad3 modulate the expression of CTSL to regulate paclitaxel resistance, and smad3 could bind to the SBE of CTSL to control the transcription of CTSL(Additional file 4: Figure S4). Egr-1 and CREB mediate the expression of CTSL to regulate cisplatin resistance, and CREB increases the transcriptional activity of CTSL by binding with CTSL promoter. Thus, CTSL may represent a novel therapeutic target for reinforcing the efficacy of cancer chemotherapy.

## Additional files

Additional file 1: **Figure S1.** CTSL is highly expressed in drug-resistant lung cancer cell lines. CCK8 was performed to determine the IC50 and RI of A549/TAX (A) and A549/DDP cells (B). Western blot and immunofluorescence analysis were adopted to determine the expression level of CTSL of A549, A549/TAX and A549/DDP cells (C and D). At least three independent experiments were performed. *P < 0.05, **P < 0.01 and ***P < 0.001 compared with control. (TIF 4678 kb)

Additional file 2: **Figure S2.** (A) A549/TAX cells were treated with SB431542, western blot detected the expression of CTSL. At least three independent experiments were performed. (B) A549 cells were treated with different concentration of cisplatin and harvested at 45 min, 1 h, 3 h and 6 h, western blot was used to detect the expression level of Egr-1. (C) A549 cells were treated with different concentration of cisplatin for 12 h, 24 h and 48 h, and western blot determined the expression level of CREB. At least three independent experiments were performed. *P < 0.05, **P < 0.01 and ***P < 0.001 compared with control. (TIF 11383 kb)

Additional file 3: **Figure S3.** The IC50 of PTX and DDP in H460 and PC9 cells. (A and C) CCK8 was used to detect the IC50 of PTX in H460 and PC9 cells. (B and D) CCK8 was used to detect the IC50 of DDP in H460 and PC9 cells. At least three independent experiments were performed. *P < 0.05, **P < 0.01 and ***P < 0.001 compared with control. (TIF 12322 kb)

Additional file 4: **Figure S4.** Schematic diagram of the mechanism by which regulates CTSL-mediated drug resistance. (TIF 2013 kb)

## Data Availability

The datasets supporting the conclusions of this article are included within the article and its additional files.

## Abbreviations

A549/DDP: Cisplatin-resistant A549 cells
A549/TAX: Paclitaxel-resistant A549 cells
CREB: cAMP-response element
CTSL: Cathepsin L
DDP: Cisplatin
Egr-1: Early growth reponse-1
EMT: Epithelial-mesenchymal transition
PTX: Paclitaxel
SBE: Smad-binding element
TGF-β1: Transforming growth factor-β1
