# Supplementary figures and images for: Cathepsin L-mediated resistance of paclitaxel and cisplatin is mediated by distinct regulatory mechanisms
Source: J Exp Clin Cancer Res. 2019 Aug 1;38:333. doi: 10.1186/s13046-019-1299-4 (PMC6670178; doi:10.1186/s13046-019-1299-4)

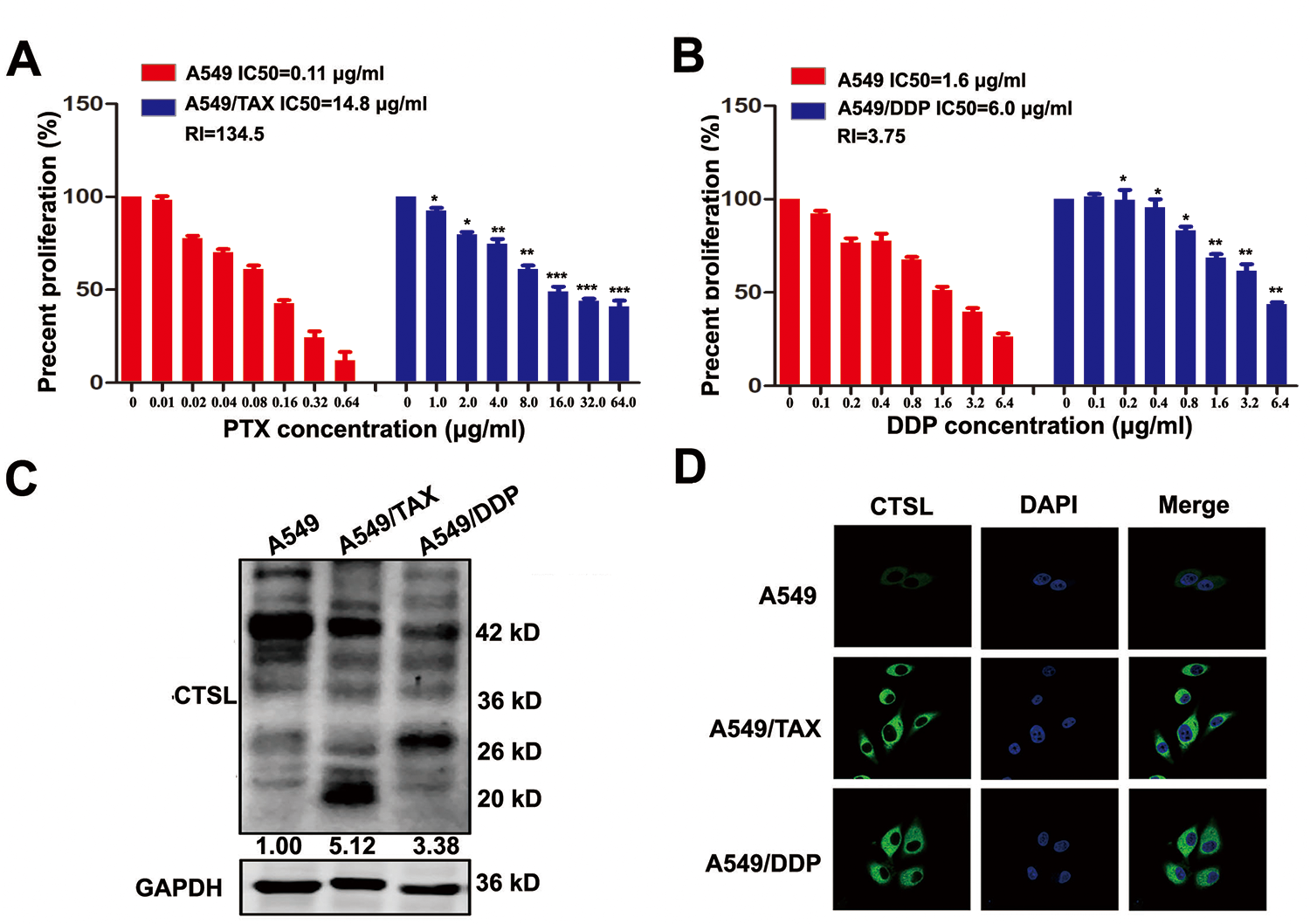

Supplement: Supplementary file 1 — Figure S1. CTSL is highly expressed in drug-resistant lung cancer cell lines. CCK8 was performed to determine the IC50 and RI of A549/TAX (A) and A549/DDP cells (B). Western blot and immunofluorescence analysis were adopted to determine the expression level of CTSL of A549, A549/TAX and A549/DDP cells (C and D). At least three independent experiments were performed. *P < 0.05, **P < 0.01 and ***P < 0.001 compared with control. (TIF 4678 kb) [file 13046_2019_1299_MOESM1_ESM.tif]

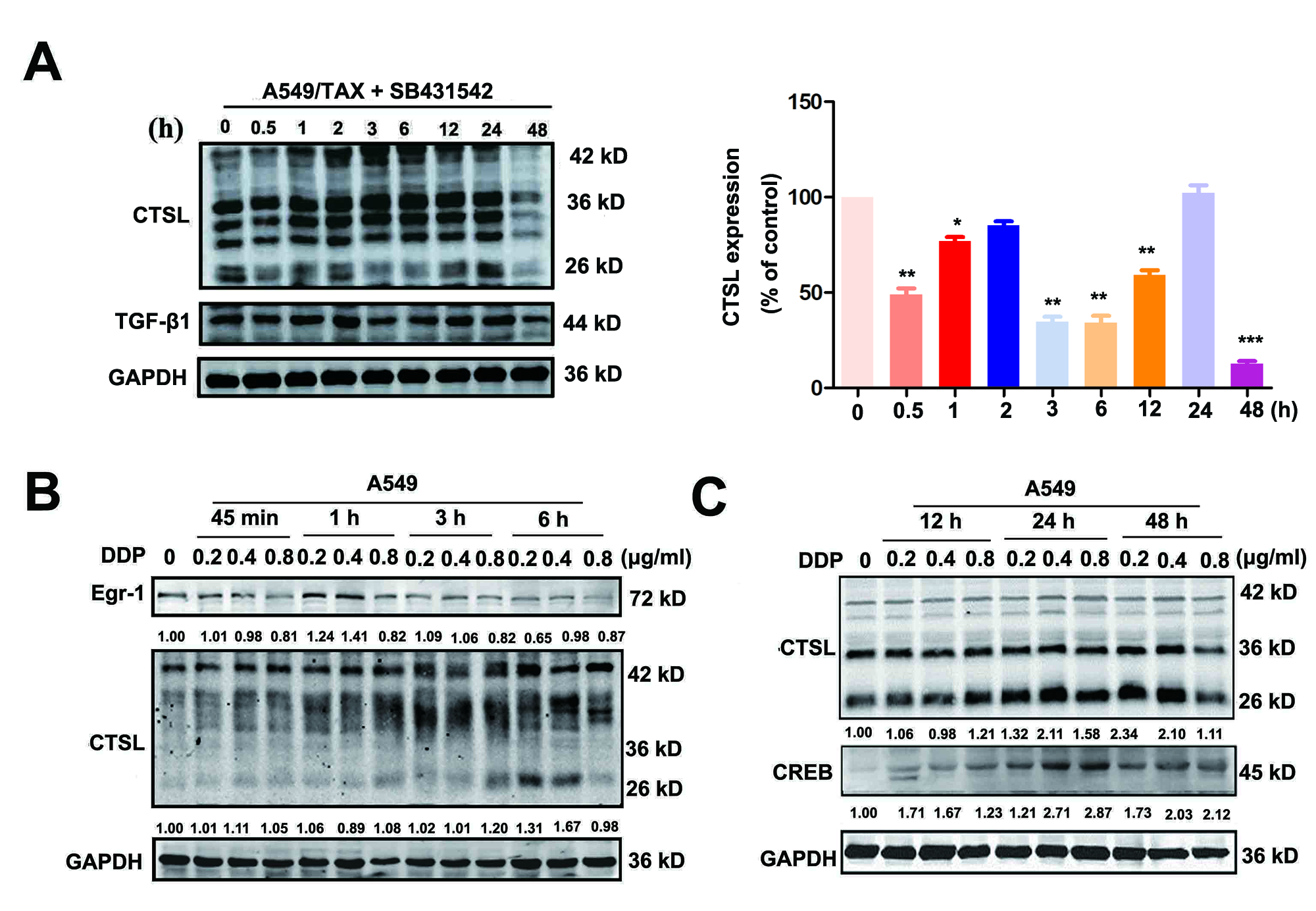

Supplement: Supplementary file 2 — Figure S2. (A) A549/TAX cells were treated with SB431542, western blot detected the expression of CTSL. At least three independent experiments were performed. (B) A549 cells were treated with different concentration of cisplatin and harvested at 45 min, 1 h, 3 h and 6 h, western blot was used to detect the expression level of Egr-1. (C) A549 cells were treated with different concentration of cisplatin for 12 h, 24 h and 48 h, and western blot determined the expression level of CREB. At least three independent experiments were performed. *P < 0.05, **P < 0.01 and ***P < 0.001 compared with control. (TIF 11383 kb) [file 13046_2019_1299_MOESM2_ESM.tif]

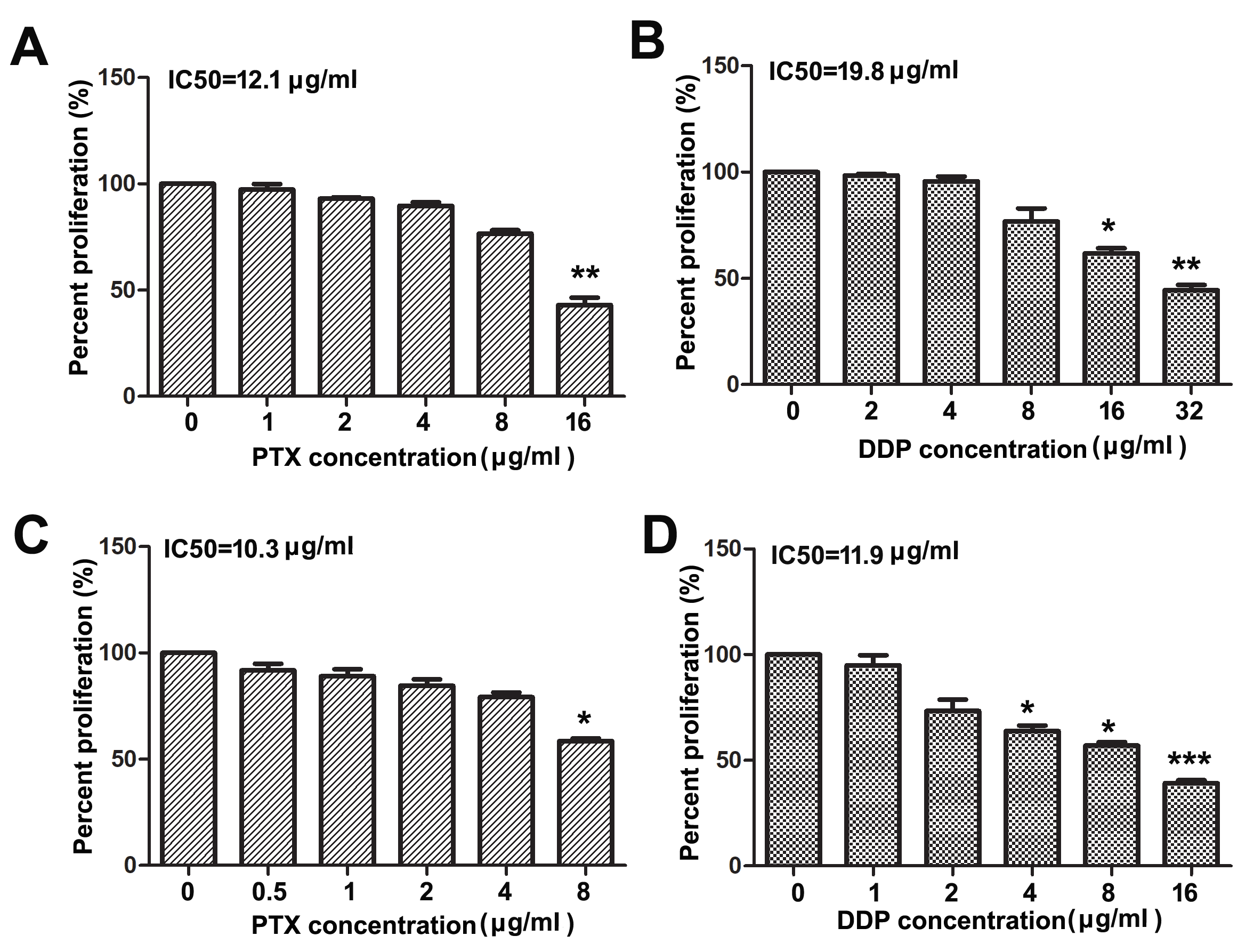

Supplement: Supplementary file 3 — Figure S3. The IC50 of PTX and DDP in H460 and PC9 cells. (A and C) CCK8 was used to detect the IC50 of PTX in H460 and PC9 cells. (B and D) CCK8 was used to detect the IC50 of DDP in H460 and PC9 cells. At least three independent experiments were performed. *P < 0.05, **P < 0.01 and ***P < 0.001 compared with control. (TIF 12322 kb) [file 13046_2019_1299_MOESM3_ESM.tif]

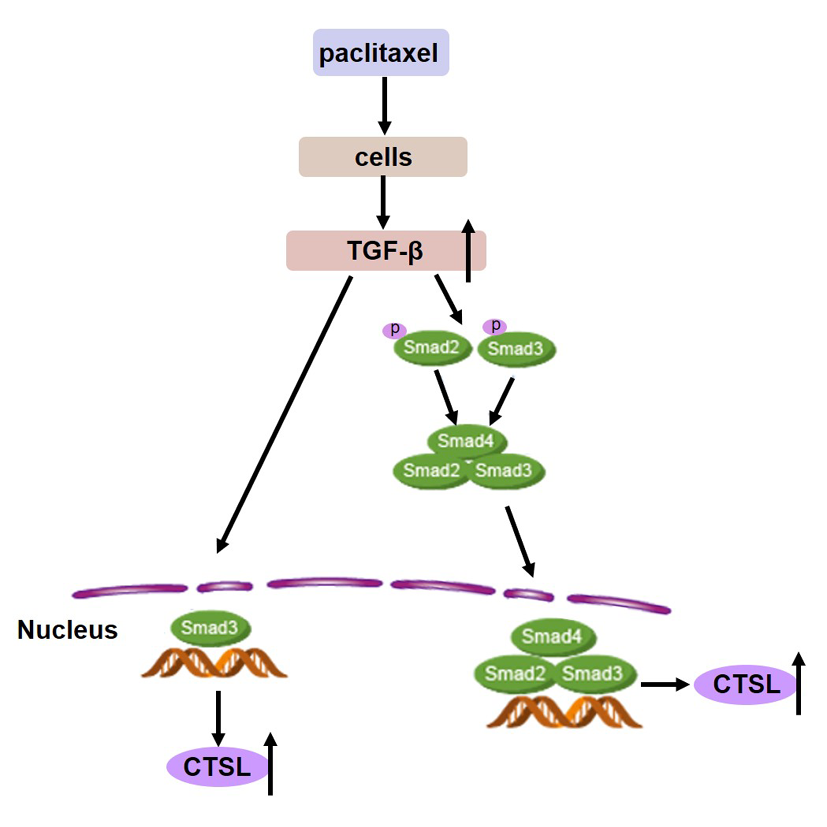

Supplement: Supplementary file 4 — Figure S4. Schematic diagram of the mechanism by which regulates CTSL-mediated drug resistance. (TIF 2013 kb) [file 13046_2019_1299_MOESM4_ESM.tif]
